# Design of a Planar Array of Low Profile Horns at 28 GHz

**DOI:** 10.3390/s20236989

**Published:** 2020-12-07

**Authors:** Jou-Yi Wang, Malcolm Ng Mou Kehn, Eva Rajo-Iglesias

**Affiliations:** 1 Department of Electrical and Computer Engineering, National Chiao Tung University, Hsinchu 30010, Taiwan; cpshnctu.cm07g@nctu.edu.tw (J.-Y.W.); malcolm.ng@ieee.org (M.N.M.K.); 2 Signal Theory and Communications Department, University Carlos III of Madrid, 28911 Leganes, Spain

**Keywords:** millimeter wave antennas, low profile horns, transverse slotted waveguide arrays, grating lobes

## Abstract

A planar array of low profile horns fed by a transverse slotted waveguide array in the low millimeter-wave regime (28 GHz) is presented. The array of transverse slots cannot be directly used as antenna as it has grating lobes due to the fact that slot elements must be spaced a guided wavelength. However, these slots can be transformed into low profile horns that with their radiation patterns attenuate the grating lobes. To this aim, low profile horns with less than 0.6$\lambda_{0}$ height were designed. The horns include a couple of chips that contribute to further reduce the grating lobes especially in the H-plane. The good performance of the designed array was demonstrated by both simulations and experiments performed on a manufactured prototype. A 5 × 5 array was designed that has a measured realized gain of 26.6 dBi with a bandwidth below 2%, still useful for some applications such as some radar systems. The total electrical size of the array is 6.63$\lambda_{0}$× 6.63$\lambda_{0}$. The radiation efficiency is very high and the aperture efficiency is above 80%. This all-metal solution is advantageous for millimeter-wave applications where losses sustained by dielectric materials become severe and it can be easily scaled to higher frequencies.

## 1. Introduction

Slotted-waveguide antenna arrays are endowed with many advantages, such as high gain, high efficiency, good polarization performance with low cross polar radiation, conformality, ease of manufacture, and thus cost effectiveness, as well as having mechanisms for the control of radiation properties (via the slot parameters and orientations) and high power handling capability. This type of antenna finds important applications in emerging technologies of the millimeter-wave (mm-wave) regime, such as 5G wireless communications, automotive and base station antennas, Internet of Things (IoT), smart cities, and autonomous radar systems [1,2,3].

Longitudinal slots on the broad-walls of rectangular waveguides have been the prevalent configuration for two-dimensional (2D) arrays. By displacing slots from the waveguide axis in a staggered manner and spacing adjacent offset slots by half the guided wavelength along the axis, broadside radiation without grating lobes can be achieved [4]. For transverse slotted waveguide arrays, however, no such simple maneuver by mere arrangements of the slots is feasible. For broadside radiation, neighboring transverse slots are compelled to be separated by one guided wavelength, leading to a unit-cell period that exceeds the free-space wavelength. As a result, grating lobes inevitably emerge. More complicated options using the narrow-walls of the waveguide have been also proposed [5].

There have been previous efforts made to alleviate the problem of grating lobes in transverse waveguide slot arrays. Baffles comprising parallel plates to suppress grating lobes were adopted by Josefsson [6]. The cancellation of reflection by using pairs of slots for transverse slot arrays was reported by Sakakibara et al. [7]. A straightforward approach of reducing the guided wavelength by partial filling of the waveguide with dielectric was studied by Joubert [8]. Haron et al. [9] used unequal lengths of non-resonant transverse slots contained within a unit cell of period less than the free-space wavelength for phase compensation. In the spirit of Sakakibara et al. [7], the reduction of reflection from the slot by the use of inductive posts was investigated by Park et al. [10]. These concepts of reflection cancellation for the mitigation of grating lobes persisted in [11,12], in which parasitic dipole layers were incorporated over the slot array.

Despite these studies, the suppression of grating lobes in transverse slotted waveguide arrays remain pertinent, especially in the wake of mm-wave architectures that impose ever-increasing demands for higher gains, channel capacities, and data rates, all of which are in conflict with the aggravated losses at those high frequencies. Many previous approaches for grating lobe reduction have involved dielectric materials, the dissipation through which is particularly severe in the mm-wave regime. This motivates alternative solutions that entail only metallic parts which do not incur dissipating losses at increased frequencies that are as grave as those suffered by dielectric components.

In some recent studies of waveguide slot arrays made entirely of metal [13,14], a one-dimensional (1D) transverse slot array on the broad-wall of a rectangular gap waveguide for broadside radiation at 28 GHz was reported, and in which grating lobe suppression was achieved by laying a corresponding 1D array of external low profile horns over the slots, constituting an all-metal topology. In other words, the slots are transformed in low profile horns whose radiation patterns act as spatial filters for the grating lobes, attenuating them. Besides, as horns are non-resonant antennas, they have advantages when used in arrays [15].

Extending the latter work, this paper investigates the design of a 2D planar array of low profile horns fed by a 2D slotted waveguide system. The design is made at 28 GHz, a frequency in the low mm-wave regime. The transformation of the slots into horns allows the suppressing of grating lobes, not only in E-plane, but in H-plane as well for the original transverse slotted waveguide starting point. A relevant remark is that, actually, in H-plane, the inter-element distance of the reference slots could be set as less than one wavelength. However, with the addition of the horns, there is higher flexibility for the separation also in this plane and in this study we decided to keep the same distance for the two planes with the purpose of obtaining an almost symmetrical radiation pattern in the two main planes. The horns are low profile ones (less than 0.6$\lambda_{0}$) and modifications of their geometry to achieve a more uniform field distribution in H-plane are presented. Designs by simulations using CST Microwave Studio^®^ (henceforth, just CST) were first performed, followed by experimental validation via measurements carried out on a manufactured prototype.

A brief outline of the paper is as follows. The designs by simulations of the feed system i.e., the 2D transverse waveguide slot array initially without the horns are described in Section 2. This is followed in Section 3 by the presentation of the horn array placed over the radiating waveguide slot array that is fed in the same way, along with simulation results that demonstrate how the designed horns can mitigate grating lobes. Measurement results of experiments carried out on a manufactured prototype according to the simulated design are given in Section 4. Finally, the paper wraps up with a conclusion that summarizes the key aspects of the work.

## 2. Design of the 2D Transverse Waveguide Slot Array

**Figure 1:**
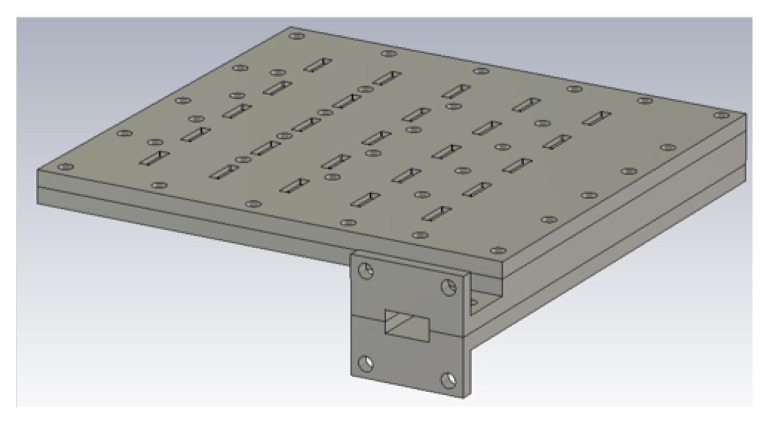
Perspective view of the designed 2D transverse waveguide slot array without horns.

As a first step, the 2D array of transverse slots is designed. The structure is used as the feed network of the array of horns. This array consists of transverse slots on the broad-walls of rectangular waveguides [16] placed side-by-side one another and fed by a single perpendicularly-oriented waveguide placed beneath (as in [17,18,19]). A 5 × 5 array is considered in this case, thus entailing five collated waveguides making up the upper layer and each with five transverse slots cut out of its upper radiating broad-wall, as well as an underlying feed waveguide with also five slots on its broad-wall spaced by a guided wavelength that couple the energy to the five upper collated radiating waveguides. A perspective view of the schematic is given in Figure 1. As explained above, the inter-slot separations in each waveguide (E-plane of the array) coincides with the guided wavelength and as a consequence exceeds the free-space wavelength. This inter-slot distance determines the size of the horns used in the array. For 2D periodicity, i.e., for the H-plane, there is a bit more of freedom to decide the distance among rows. However in this case, we decided to feed the structure with an identical waveguide, i.e., the feed waveguide underneath with transverse slots that is used to feed the five waveguides also requires an inter-element distance of one guided wavelength. Obviously, a change in the feeding waveguide width could have permitted a closer separation of the slotted rows. In summary, the inter-slot separations are identical in E- and H-planes in this design. This decision conditions the size of the horns, in this case making them squared.

**Figure 2:**
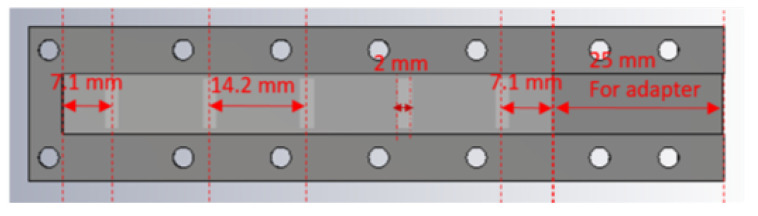
Interior of the feeding waveguide.

**Figure 3:**
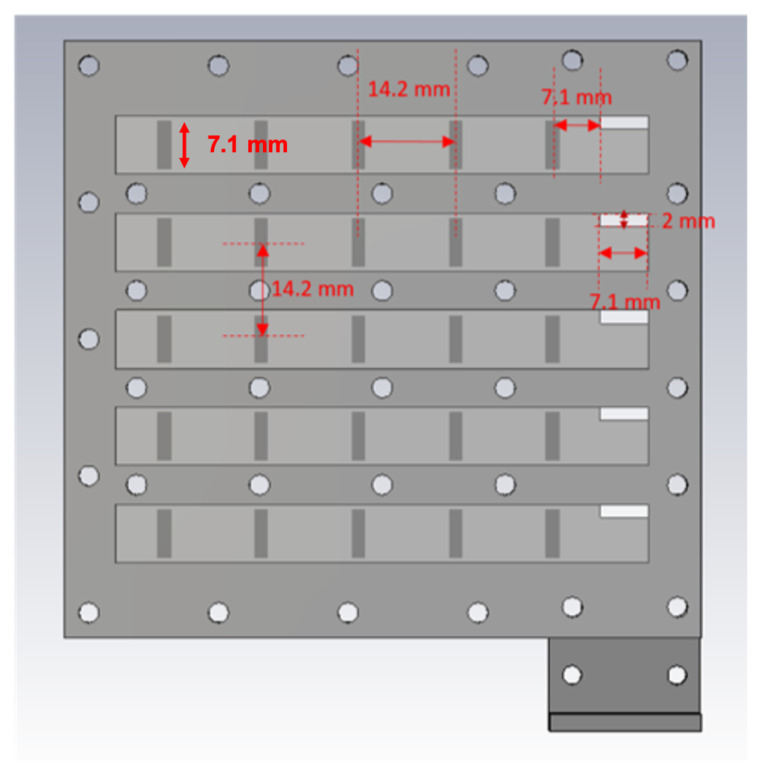
Interior of the radiating waveguides.

The feeding structure is designed in a classical way. It starts with the selection of the waveguide, in this case a standard WR-34 waveguide with width a = 8.6 mm and height b = 4.3 mm whose recommended frequency is from 22.00 to 33 GHz, thereby suiting the presently prescribed 28 GHz operating frequency. The size of each radiating slot is set as 7.1 mm × 2 mm after initial design by simulations. As mentioned, located on the upper broad-wall of the underlying waveguide are five feeding slots that couple to the upper layer of laterally arranged set of waveguides via one of their terminal ends. Twenty-five radiating slots are located on the collective radiating planar broad-walls of the collated waveguides. The $TE_{10}$ modal guided wavelength is 13.7 mm at 28 GHz for the width of 8.6 mm. However, upon visual inspection of the simulated interior field distribution along the waveguide axis, it is observed that one field cycle (guided wavelength) is about 14.2 mm, being a slight shift from the theoretically expected value. This minor shift in guided wavelength is due to the deviation of the finite structure simulated in the CST software from the infinitely long waveguide assumed in theory. Hence, the inter-slot separation implemented in the simulations is set as 14.2 mm in both planes, and the distance of the terminal slot to the end wall of the waveguide is set as 7.1 mm, being half the guided wavelength. A view of the feed waveguide seen from the inside is given in Figure 2, which reveals the array of transverse slots on the outer side displayed in lighter shade. The circles are just holes for fixing the top layer of the prototype. The total length of the interior of the feeding waveguide is 96 mm, which includes the 25- mm length of the adapter. The radiating waveguides are placed above the feeding slots, as shown in Figure 3, in which the placements of these latter relative to the fed ends of the former are portrayed.

As shown in Figure 3, the feeding slots are located at one side of the radiating waveguides rather than in the middle. The reason for this is better compatibility between the fields in the feeding slots and the fields in the upper waveguides, being an outcome based on a thorough process of design by simulations of the feeding waveguide.

This structure was simulated and optimized as an antenna, although, in the next section, the slots are transformed into low profile horns. However, the design of this 2D planar slot array guarantees the excitation of the 25 slots (and, as a consequence, also the 25 horns) with equal amplitude and phase. The structure is well matched and if we observe the radiation patterns we could see the grating lobes in the two main planes. These results are omitted here but are presented below in the experimental verification of the antenna.

For the calculation of the directions of the grating lobes, we must consider that the elevation angle θ is measured from the z axis, which, for the present case, is along the axes of the waveguides. For desired broadside main beam with $\theta_{0}$= 90°, the subscript 0 denoting the dominant 0th-order Floquet harmonic, these nearest-in undesired lobes appearing in visible space show up towards the directions predicted by the following relation:

(1) $$\theta_{\pm 1}=90^{\circ }\pm \sin^{-1}\frac{2\pi /p}{\kappa_{0}}$$

where p = 14.2 mm is the simulated period (or inter-slot spacing along the waveguide axis), $\kappa_{0}$ = $2\pi f/c_{0}$ is the free-space wavenumber, and f is the frequency. The ±1 denotes the two higher ordered Floquet harmonics. At f = 28 GHz, the two grating lobes are towards θ = 90°± 49° = 41° and 139° in the two main planes.

## 3. Design of the Horn Structure

**Figure 4:**
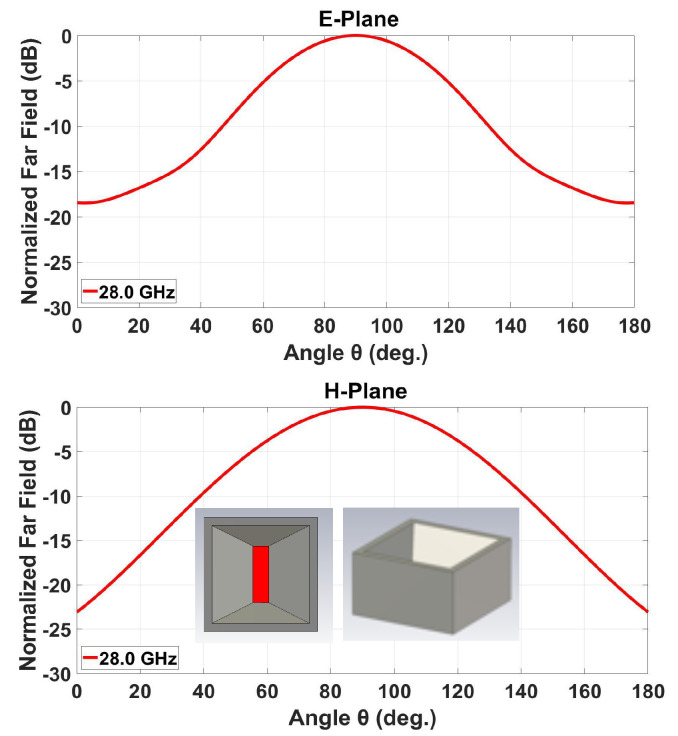
Simulated radiation patterns of single ordinary horn element (without pair of metal chips) in both principal planes: (**top**) E-plane perpendicular to slot alignment; and (**bottom**) H-plane parallel to slot alignment. Horn schematics (two views) are shown as inset diagrams.

After designing the 2D slotted array, it was used as the feed system for the horns. The rectangular throat aperture of these pyramidal horn elements takes on the same dimensions as the transverse slot on the broad-wall of the rectangular waveguide, being 7.1 mm × 2 mm as stated above. This slot flares linearly to a square outermost radiating horn aperture of size 12.2 mm × 12.2 mm. The height of the radiating horn in this case is set to 7.1 mm to keep it low profile following the preliminary design in [13]. For a single such horn, the simulated radiation patterns in both principal planes are given in Figure 4, inset diagrams within which depict the structure. As calculated, according to (1), the two grating lobes of the array are towards 41° and 139° in both planes. As shown in Figure 4, the anticipated attenuation of these lobes in E-plane is about 12.5 dB and around 10 dB in H-plane. This means that the suppressing of grating lobes in E-plane is more effective than in H-plane. The reason is that the horn has a uniform field distribution in the E-plane but a cosine type one in H-plane. As a consequence, the radiation pattern is less directive in H-plane. Further remedial design maneuvers are thus required.

**Figure 5:**
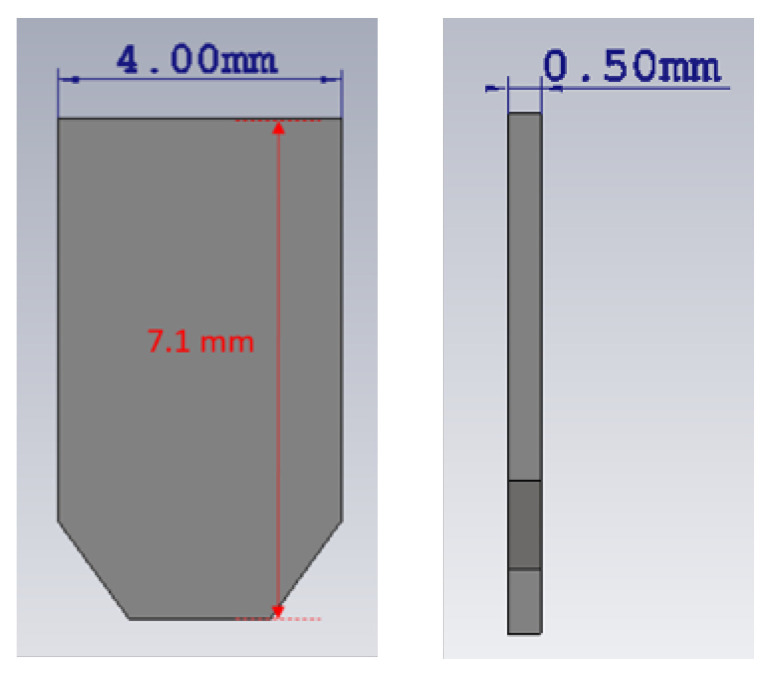
Size of the chips.

**Figure 6:**
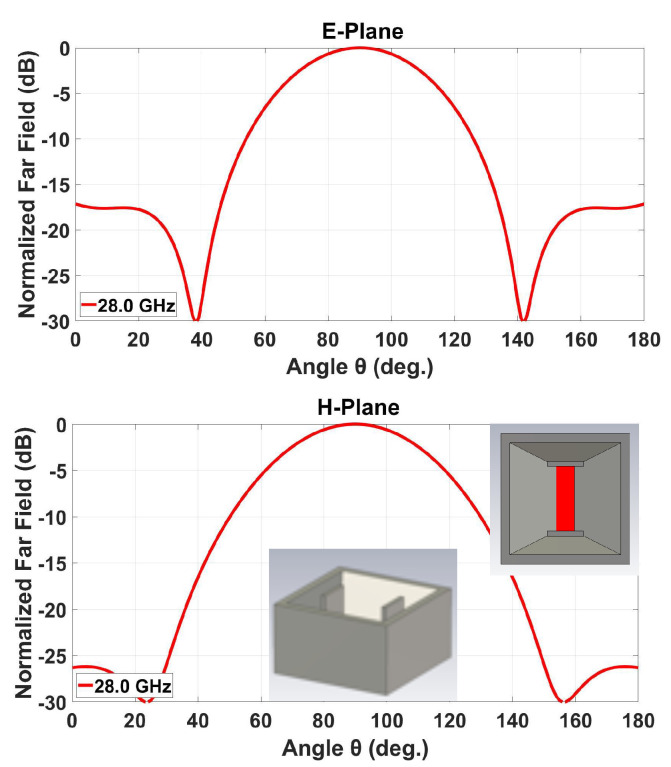
Simulated radiation patterns of single modified horn element with inserted pair of metal chips in both principal planes: (**top**) E-plane perpendicular to slot alignment; and (**bottom**) H-plane parallel to slot alignment. Horn schematics (two views) are shown as inset diagrams.

Inspired by the baffles proposed in [6], a pair of parallel vertically-oriented rectangular metal chips, as described in Figure 5, is inserted into each horn, which are depicted by the inset diagrams of Figure 6. The plots in the latter present the simulated radiation patterns in both principal planes of this modified horn element. Each chip has a height of 7.1 mm, a width of 4 mm, and a thickness of 0.5 mm, as shown in Figure 5.

For the same two grating lobe directions of θ= 41° and 139°, the projected attenuation of these lobes are shown in Figure 6 to be about 30 dB in E-plane and around 17 dB in H-plane, which are considerable improvements over the initial horn.

**Figure 7:**
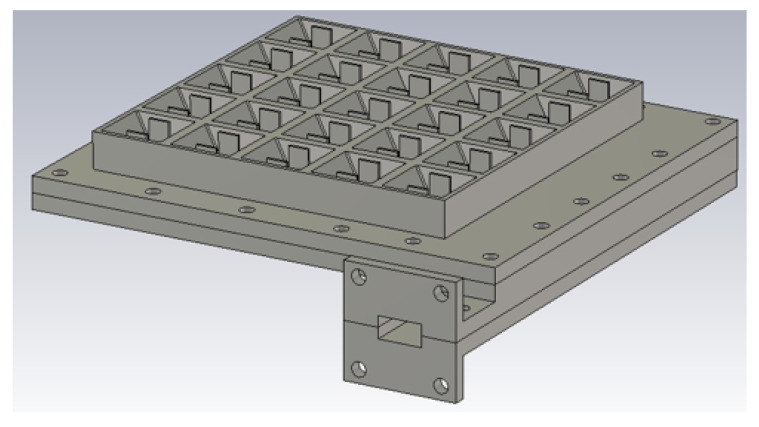
Schematic of the designed structure with horns.

**Figure 8:**
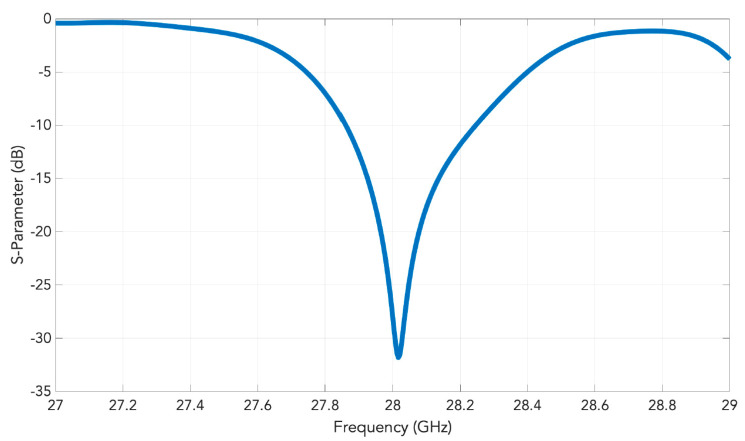
Simulated $S_{11}$ of the array of horns fed by the 2D transverse waveguide slot array.

**Figure 9:**
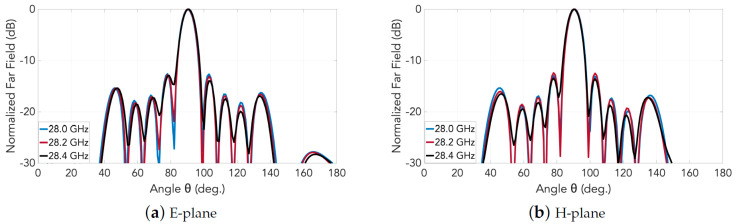
Simulated far-field radiation patterns of the 2D transverse waveguide slot array with horns.

**Figure 10:**
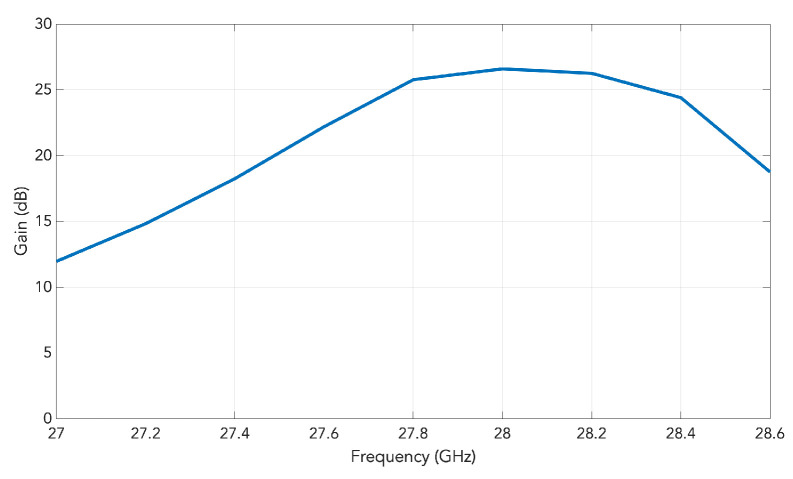
Simulated realized gain of the array of horns fed the 2D transverse waveguide slot array as a function of the frequency.

A schematic of the entire 2D transverse waveguide-slot array covered by a planar array of such special pyramidal horn elements with metal chips is illustrated by Figure 7. For this whole structure, the simulated response of the $S_{11}$ is presented in Figure 8, from which the operating bandwidth is seen to be 27.860–28.246 GHz. The simulated E-plane radiation patterns are provided in Figure 9a and those in H-plane are given by Figure 9b. The grating lobes that would have been present are now effectively suppressed in both principal planes by the 2D array of external horns, each with a pair of chips, by about 15 dB in E-plane and 10 dB in H-plane at the 28 GHz design frequency. For the waveguide with horns, the simulated beamwidth is about 7.6° in E-plane and 7.5° in H-plane at the 28 GHz design frequency. The radiation pattern is quite symmetrical. The simulated realized gain is shown in Figure 10 with a maximum of almost 26.6 dBi.

### Operation Principle

The fundamentals of the behavior of the new horns and how they deal with the grating lobe attenuation can be explained in different ways. The addition of the chips clearly contributes to make more uniform the aperture field distribution in H-plane, and as a consequence the horn with chips has a directivity of 13 dBi, while, without chips, the directivity is 11.5 dBi. This difference is gained by this design in aperture efficiency. As a consequence, when used in an array, applying the principle of multiplication of patterns a higher reduction of the grating lobe will be obtained.

There is another option to explain this improvement by considering the array of horns with and without chips and observing their aperture fields. The added pair of parallel metal chips within each horn, whose separation $d_{chip}$ = 7.1 mm displacement (perpendicular to the chips) is along the H-plane, produces the effect of reducing the effective electrical (rather than physical) periodicity along the H-plane of the fields on the radiating aperture, to one that equals 7.1 mm, being half of the physical unit-cell size of 14.2 mm along the H-plane of the structure. The distance between the chips within the same horn is 7.1 mm and that between two adjacent chips but inside neighboring horns is also 7.1 mm. This halved period then leads to the mitigation of grating lobes in this plane. A similar approach by using a septum was proposed by Vosoogh et al. [20]. The simulated realized gain of the array of horns fed by the 2D transverse waveguide slot array as a function of the frequency is presented in Figure 10.

**Figure 11:**
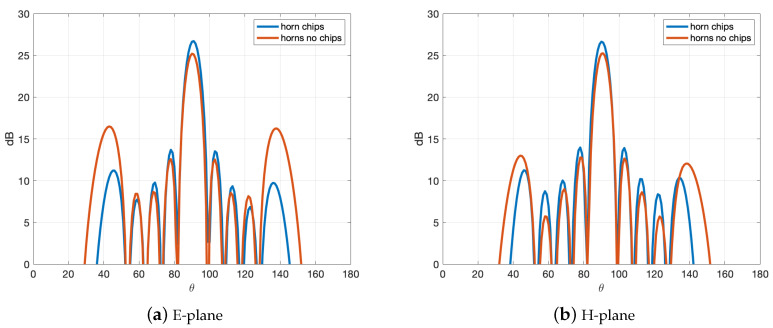
Simulated far-field directivity of the array of horns with and without chips, both fed by the 2D transverse waveguide slot array.

Finally, a comparison of the array with the designed horns with chips with an identical array but with the conventional low profile horns without chips is presented in Figure 11. The increase in the directivity motivated by the evident decrease on the grating lobe levels can be clearly observed in the figures.

## 4. Experimental Results

**Figure 12:**
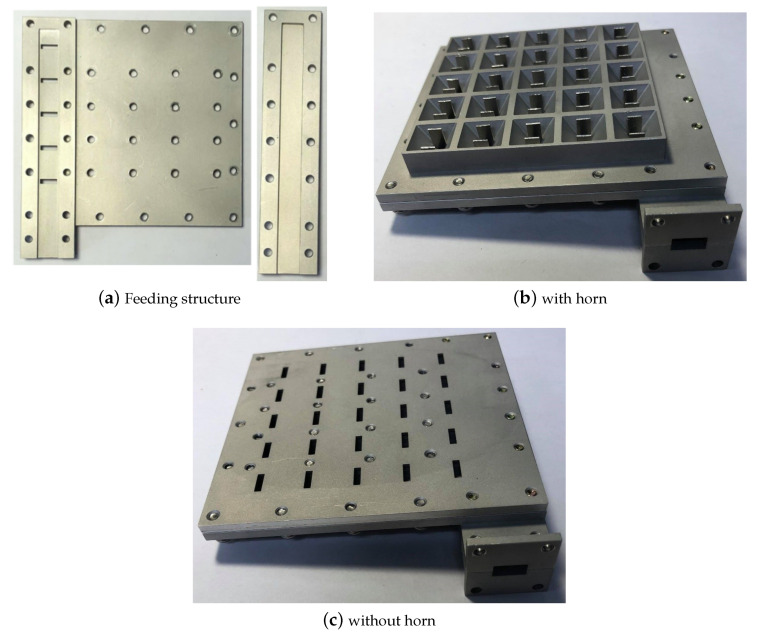
Photographs of the manufactured prototype.

In this section, the experimental results of the array of horns which is the focus of this work are presented. However, we also manufactured and measured one layer corresponding to the planar slot array, as we can also consider this paper as a contribution to reduce grating lobes in transverse-slotted waveguide. Besides, the methodology of design of our proposed antenna, i.e., the array of low profile horns, has as a first step the design of the 2D slotted waveguide. The feed waveguide is a shared component between the two prototypes, which itself is constructed as a lower and an upper piece, as photographed in Figure 12a, which are screwed together via the sidewalls, a procedure typical of waveguide fabrication. For each of the topologies (just slots and horns), the upper layer comprising the collated slotted waveguides is likewise fabricated as two separate entities, a lower piece and an upper structure, attachable to each other by screws as well. Clearly, as with the feed waveguide, the lower portion is also common to both configurations. It is the upper part that is distinct between the two: one for the case of horns, as photographed in Figure 12b, and another for the slots, as seen in Figure 12c.

**Figure 13:**
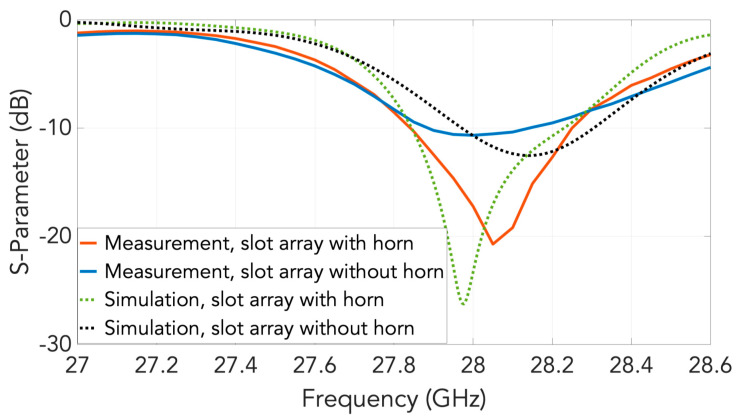
Measured and simulated $S_{11}$ of the 2D transverse waveguide slot array with and without horns.

The measured $S_{11}$ of the manufactured prototype with slots and with horns are presented in Figure 13 over a band centered at the prescribed 28 GHz, alongside the corresponding simulated results. Evidently, the designated operation band is realized in the experiments.

**Figure 14:**
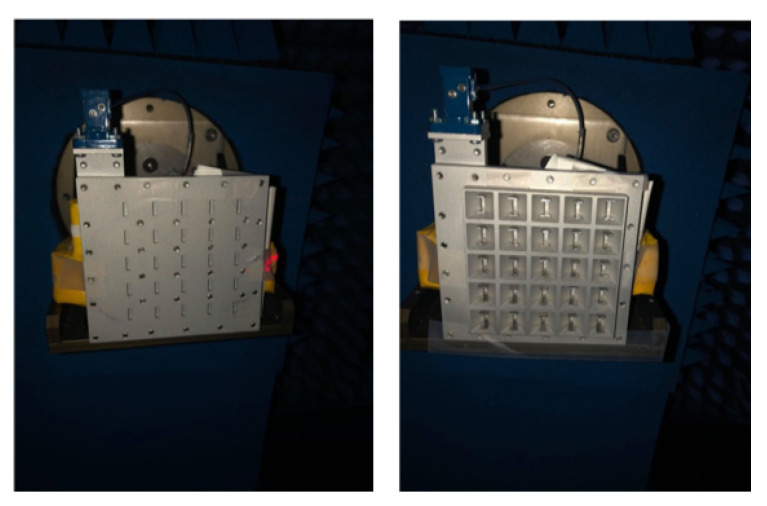
Photographs of experimental setup in anechoic chamber for measurements of far-field radiation patterns: (**left**) without horns; and (**right**) with horns.

**Figure 15:**
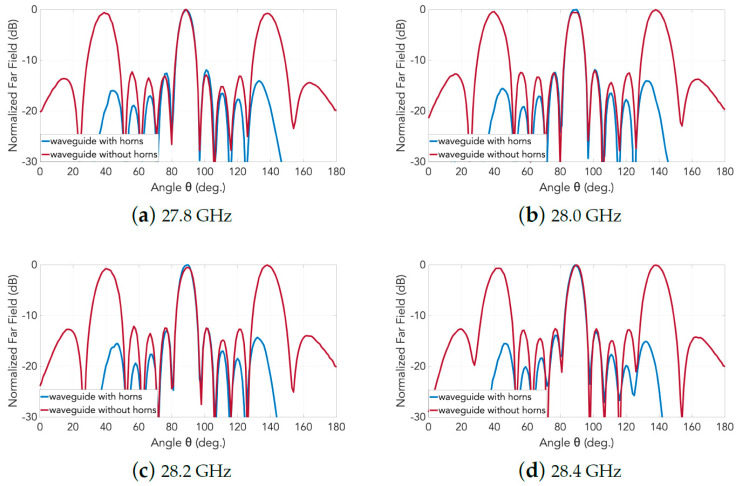
Measured normalized radiation patterns in E-plane of the 2D transverse waveguide slot array with and without horns: (**a**) 27.8 GHz;(**b**) 28.0GHz; (**c**) 28.2 GHz; and (**d**) 28.4 GHz.

**Figure 16:**
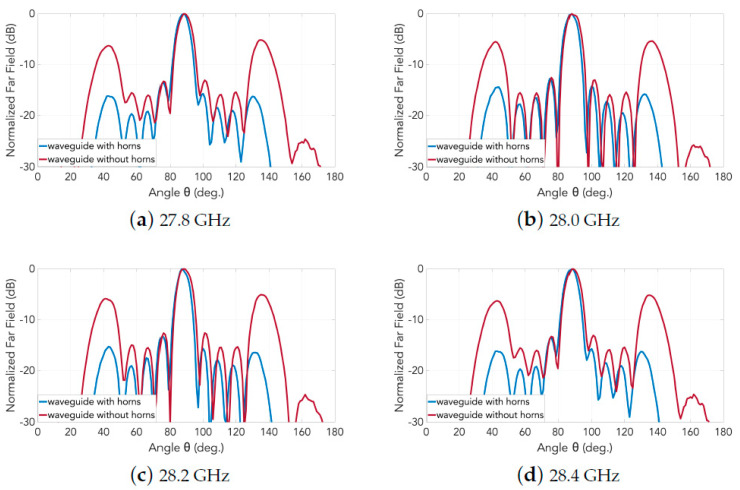
Measured normalized radiation patterns in H-plane of the 2D transverse waveguide slot array with and without horns: (**a**) 27.8 GHz; (**b**) 28.0 GHz; (**c**) 28.2 GHz; and (**d**) 28.4 GHz.

Obtained from experiments carried out in an anechoic chamber, photographs of which are shown in Figure 14, the measured far-field normalized radiation patterns in E- and H-planes are presented, respectively, in Figure 15 and Figure 16. Any one subplot within every figure pertains to a certain frequency as labeled. Two traces are given in each graph, one for the case of the horns and the other for just the slots (named as “without horns”), thereby portraying the effective grating lobe suppression at every frequency for both principal planes.

**Figure 17:**
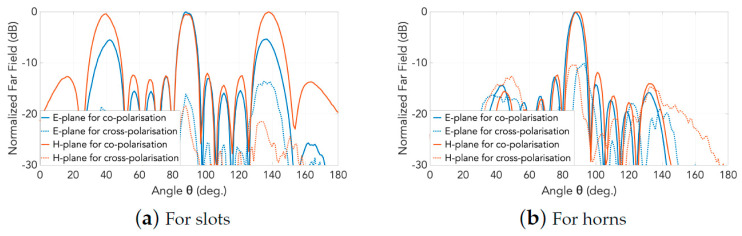
Normalized measured far-field patterns (solid traces, co-polar; dotted traces, cross-polar): (**a**) for slots; and (**b**) for horns.

For cases with and without horns, the measured co- and cross-polar radiation patterns in the central frequency are presented in Figure 17. As can be seen, the cross polar levels towards the main broadside beam direction are raised to about –10 dB, which is due to the modifications of the horns.

**Figure 18:**
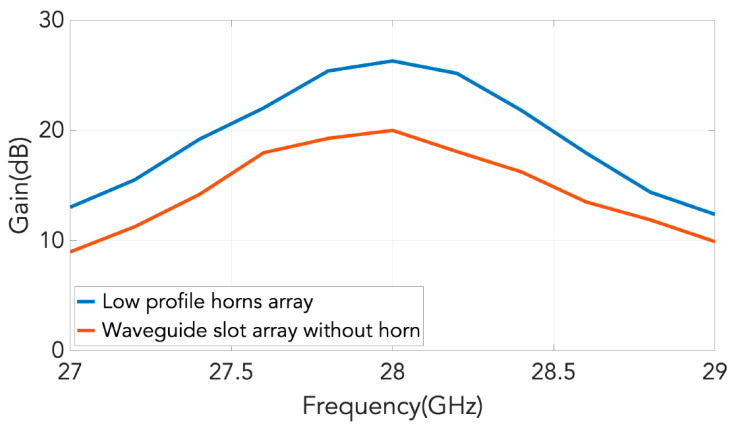
Measured gains of the manufactured 2D transverse waveguide slot array with and without horns.

The measured realized gain of the designed antenna is shown in Figure 18. In the same graph, the measured gain of the array of slots is also represented to show how the reduction of the grating lobes is translated in an average increment of about 7.5 dB in gain over the considered band. The maximum measured realized gain for the array of low profile horns is 26.3 dB and the simulated directivity is 26.65 dB. The beamwidth of the measured radiation patterns for the waveguide with horns is about 8 degrees in E-plane and 8.7 degrees in H-plane at the prescribed 28 GHz operating frequency, keeping the symmetry observed in simulations.

Finally, it is worth mentioning that the measured grating lobe suppression (compared to the case of the array of slots) in both principal planes is about 15 dB in E-plane and 9 dB in H-plane in the entire frequency range.

### 4.1. Antenna Efficiency

Radiation efficiency is typically defined as the ratio between the measured realized gain and the simulated directivity. In this way, it is a term that accounts for the antenna ohmic losses (dissipation) and mismatch (reflection) losses.

However, when dealing with aperture antennas, another efficiency that is often used is the aperture efficiency. Ideally, this efficiency is the ratio between the antenna directivity and the maximum theoretical directivity that one antenna with its size can achieve, i.e.,

$$D_{max}=\left(4\pi /\lambda^{2}\right)S$$

where *S* is the physical surface of the antenna.

The aperture efficiency is then defined as:

$$\epsilon_{ap}=D_{0}/D_{max}$$

where $D_{0}$ accounts for the classical definition of maximum directivity.

**Figure 19:**
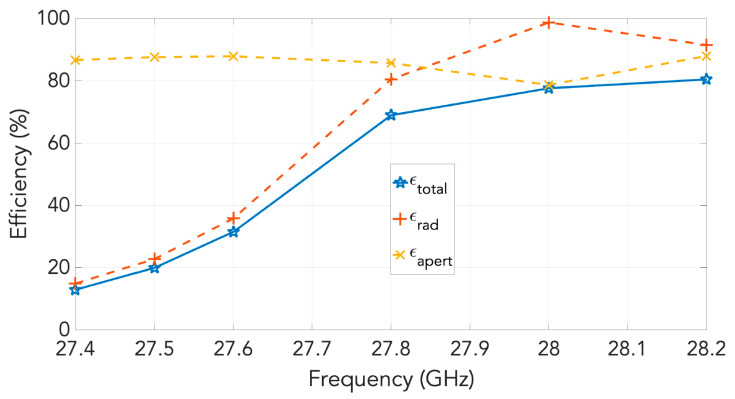
Aperture efficiency and radiation efficiency as a function of the frequency for the low profile horn array.

Following these definitions, we calculated these two efficiencies, and they are represented in Figure 19. Here, we also define another efficiency named as “total efficiency” that is the product of the other two. The graph of the various sub-efficiencies versus frequency for our antenna array is provided in Figure 19. At 28 GHz, the achieved approximate 80% total efficiency is about −1 dB, and, because the radiation efficiency that is −0.2 dB at 28 GHz is very high, this total efficiency is also almost the same as the aperture efficiency at that frequency. In other words, the radiation efficiency of this antenna is really high and the aperture efficiency is also good.

When compared to the most similar work, i.e., that of Pucci et al. [21], there, an aperture efficiency of −2.6 dB was reported together with a −1.5 dB of radiation efficiency. The design proposed in this paper clearly outperforms that one in both efficiencies despite having an operation frequency that is almost three times the one in [21].

### 4.2. Discussion

The proposed array of low profile horns can be seen as a new contribution following two main aspects. On the one hand, this can be seen as a technique to reduce grating lobes in transverse slotted waveguide array and this is the reason we show some results of the array of just slots. Even if this topic does not have a high impact, it is still deserving of some attention and that can be seen as one motivation for this contribution. Some examples of techniques to reduce grating lobes can be found in [22,23,24].

On the other hand, this is a good antenna design using low profile horns. There are not many examples of low profile horns (with less than one wavelength height) in the literature (e.g., [25,26]) or something that is not so low in profile but compact (e.g., [27]). These few existing low profile horns are not used in an array. The only example that we have found of a planar array of low profile horns is in [21], which is actually the starting point of this work. The designed array here has both a higher aperture efficiency and a higher radiation efficiency than that in [21] even considering that the frequency here is almost three times higher. The innovations with respect to the work of Pucci et al. [21] come from the new type of horns that we designed with better aperture efficiency and also from the type of feed network that we used (a simpler waveguide based feed network compared to an inverted microstrip gap waveguide) with the purpose of minimizing the feed network losses. In addition, a linear array of low profile horns (without chips) fed by a transverse slot array was presented by Ng Mou Kehn and Rajo-Iglesias [14]. However, the horns were not optimized to maximize their directivity and now in the present work, and the addition of the chips allow a further reduction in the grating lobes. A comparison summary with some related works is presented in Table 1.

As a drawback, the bandwidth is quite limited as it was not the focus of the study, and slotted waveguide antennas are inherently narrow band. Similar fractional bandwidth percentages can be found in many designs in the literature (see, e.g., [28,29,30]). However, radio communication systems with narrow band properties are not so rare, as it is the case of radar systems. Nevertheless, techniques to improve the bandwidth such as changing the shape of the slots or feeding the waveguides in the middle could always be applied [31,32].

## 5. Conclusions

A planar array of low profile horns is presented. The design is based on the use of a feed network that is a planar array of transverse slots in rectangular waveguide. These slots are transformed in low profile horns (with less than 0.6$\lambda_{0}$ height), which are specially designed by making use of a couple of chips, to have more directivity and increase the aperture efficiency of the antenna keeping the low profile characteristic. The use of transverse slots is a key point of this design as they perfectly fit for feeding the horns (it is difficult to imagine a feeding system based on the use of longitudinal slots), and, at the same time, as they have to be separated a distance equal to the guided wavelength, it allows the use of horns with a relatively big transverse section.

Designs by simulations led to the manufacture of a prototype, the measurement results of which agree well with theory, in terms of the operation band, gain, and radiation patterns. The beamwidths of the simulated radiation patterns are about 7.5° in both principal planes and around 8° in measurements. The radiation pattern is quite symmetrical.

The measured gain is above 26 dB and the antenna efficiency is very high. Due to the modifications of the horns, the cross polar levels towards the broadside main beam direction are slightly increased, which is understandable.

The focus of the study was the design of the array of horns with a feed network made with transverse slots, however this can also be seen as a technique for mitigation of the grating lobe levels in transverse slotted waveguide arrays. In this case, the grating lobes are attenuated by about 15 dB in E-plane and around 9 dB in H-plane, which has as a consequence an enhancement on the gains of the broadside main beams by about 7–7.5 dB, as shown in this study.

This proposed 2D transverse waveguide slot array composed entirely of metal and without any dielectric parts can be applied advantageously to various emerging millimeter-wave technologies. The number of slots can be easily extended to any arbitrary number and the design can be easily scaled to higher frequencies as it is made fully with metal.

## Figures and Tables

**Table 1:** Comparison of performances with related works.

REF.	Center Frequency,	Undesired Lobe	Antenna Efficiency
	Fractional Bandwidth	Suppression (dB)	(dB)
[22]	no info, 3.4%	8.1	−2.08
[24]	76.5 GHz, 1.3%	13.5	no info
[21]	10.6 GHz, 11%	8	−4
[14]	28.175 GHz, 3%	12	−2.46
Ours	28 GHz, 1.38%	15	−1.038
